# Comparing Graph Sample and Aggregation (SAGE) and Graph Attention Networks in the Prediction of Drug-Gene Associations of Extended-Spectrum Beta-Lactamases in Periodontal Infections and Resistance

**DOI:** 10.7759/cureus.68082

**Published:** 2024-08-29

**Authors:** Johnisha Harris, Pradeep Kumar Yadalam, Raghavendra Vamsi Anegundi, Deepavalli Arumuganainar

**Affiliations:** 1 Periodontics, Saveetha Dental College and Hospitals, Saveetha Institute of Medical and Technical Sciences, Saveetha University, Chennai, IND

**Keywords:** antimicrobial resistance, gene, drug, beta-lactamase, graph neural networks

## Abstract

Introduction: Gram-negative bacteria exhibit more antibiotic resistance than gram-positive bacteria due to their cell wall structure and composition differences. Porins, or protein channels in these bacteria, can allow small, hydrophilic antibiotics to diffuse, affecting their susceptibility. Mutations in porin protein genes can also impair antibiotic entry. Predicting drug-gene associations of extended-spectrum beta-lactamases (ESBLs) is crucial as they confer resistance to beta-lactam antibiotics, challenging the treatment of infections. This aids clinicians in selecting suitable treatments, optimizing drug usage, enhancing patient outcomes, and controlling antibiotic resistance in healthcare settings. Graph-based neural networks can predict drug-gene associations in periodontal infections and resistance. The aim of the study was to predict drug-gene associations of ESBLs in periodontal infections and resistance.

Methods: The study focuses on analyzing drug-gene associations using probes and drugs. The data was converted into graph language, assigning nodes and edges for drugs and genes. Graph neural networks (GNNs) and similar algorithms were implemented using Google Colab and Python. Cytoscape and CytoHubba are open-source software platforms used for network analysis and visualization. GNNs were used for tasks like node classification, link prediction, and graph-level prediction. Three graph-based models were used: graph convolutional network (GCN), Graph SAGE, and graph attention network (GAT). Each model was trained for 200 epochs using the Adam optimizer with a learning rate of 0.01 and a weight decay of 5e-4.

Results: The drug-gene association network has 57 nodes, 79 edges, and a 2.730 characteristic path length. Its structure, organization, and connectivity are analyzed using the GCN and Graph SAGE, which show high accuracy, precision, recall, and an F1-score of 0.94. GAT's performance metrics are lower, with an accuracy of 0.68, precision of 0.47, recall of 0.68, and F1-score of 0.56, suggesting that it may not be as effective in capturing drug-gene relationships.

Conclusion: Compared to ESBLs, both GCN and Graph SAGE demonstrate excellent performance with accuracy, precision, recall, and an F1-score of 0.94. These results indicate that GCN and Graph SAGE are highly effective in predicting drug-gene associations related to ESBLs. GCN and Graph SAGE outperform GAT in predicting drug-gene associations for ESBLs. Improvements include data augmentation, regularization, and cross-validation. Ethical considerations, fairness, and open-source implementations are crucial for future research in precision periodontal treatment.

## Introduction

Periodontal disease is a chronic inflammatory disease caused by the growth of periodontopathogens such as *Porphyromonas gingivalis, Fusobacterium nucleatum,* etc. in the form of biofilm, leading to the destruction of periodontal tissues and eventually leading to tooth loss. Genetic, environmental, and behavioral factors influence the severity of the disease. Periodontal pockets, which are pathologically deepened gingival sulcus, can cause infections in various systems such as cardiovascular, respiratory, etc., especially in hospitalized, elderly, and immunocompromised individuals [1]. Pathogens responsible for the initiation and progression of periodontal disease are usually treated with antibiotics such as penicillin, cephalosporins, etc. These antibiotics, when used for a longer period of time, are prone to developing resistance. Penicillin resistance can occur through three mechanisms: reduced permeability of the bacterial cell to the antibiotic, alteration of penicillin-binding proteins (PBPs), and bacterial production of inactivating enzymes called β-lactamases [2].

Penicillins are bactericidal drugs that inhibit the synthesis of the bacterial peptidoglycan cell wall, providing rigid stability to the cell. It is the only natural penicillin used clinically, while penicillin V is more stable orally [3]. Semi-synthetic penicillins have enhanced antimicrobial properties, increased stability in oral and stomach acids, and higher absorption, requiring lower dosages for therapeutic levels. The use of antibiotics has led to the development of antibiotic-resistant pathogens, posing a threat to effective disease treatment [4]. Gram-negative organisms show resistance due to the differences in the structure and composition of their cell walls compared to Gram-positive organisms. Mutations in the genes coding for porin proteins in the outer membrane of Gram-negative bacteria can impair the entry of certain antibiotics into the cell. Additionally, microorganisms may resist penicillin due to structural differences in the PBPs that target the drug [3,4].

Predicting drug-gene associations of extended-spectrum beta-lactamases (ESBLs) is crucial as they confer resistance to beta-lactam antibiotics, challenging the treatment of infections. Predicting drug-gene associations helps identify potential drugs to target ESBLs and overcome resistance mechanisms. This information guides the development of new antibiotics and repurposes existing ones. Understanding drug-gene associations also aids in optimizing drug usage and treatment strategies, reducing treatment failure, and improving patient outcomes. Predicting drug-gene associations of ESBLs is crucial for developing diagnostic tools to identify and treat resistant bacteria. This aids clinicians in selecting suitable treatments, optimizing drug usage, enhancing patient outcomes, and controlling antibiotic resistance in healthcare settings [5].

Cytoscape is an open-source software platform that enables biological network visualization, analysis, and modeling. It offers a user-friendly interface and a variety of plugins for network analysis. CytoHubba, a plugin, helps identify key nodes or genes based on topological properties [6,7].

Graph neural networks (GNNs) are deep learning models for graph-structured data, focusing on tasks like node classification, link prediction, and graph-level prediction. Common GNN architectures include graph convolutional network (GCN), Graph SAGE (sample and aggregation), and graph attention network (GAT), which use attention mechanisms for dynamic information propagation. In this study, three different graph-based models were used: GCN, Graph SAGE, and GAT. These models are designed to work with graph-structured data, representing the data as nodes connected by edges [8,9]. 

Graph SAGE is a graph-based neural network architecture designed for node classification tasks and uses an input graph with nodes representing drugs and genes and edges representing interactions between them. GAT is a neural network architecture for node classification and link prediction tasks. It uses an input graph with nodes representing drugs and genes and edges representing interactions between them [10,11]. This study aims to predict drug-gene associations of extended-spectrum beta-lactamases in periodontal infections and resistance by combining graph sage and graph attention networks which will help in framing an appropriate antibiotic treatment regimen for periodontitis patients.

## Materials and methods

Data preparation

Using probes and drugs [6], drug genes of an extended spectrum of beta-lactamases were retrieved, and this data consists of drugs and genes. Data were converted into graph language data with nodes and edges and assigned for drugs and genes as a node, target type as edge, activity biochemical as edge weight, and other columns as node features and were subjected to GNNs and their similar algorithms with the type of interaction as target using Google Colab, with python environment, these algorithms were implemented for model accuracy.

Cytoscape and CytoHubba

In Cytoscape, we uploaded to visualize the network, customize node and edge attributes, and adjust the layout. CytoHubba offers methods like degree centrality, betweenness centrality, and closeness centrality to identify critical nodes. It ranks nodes based on their importance. Advanced clustering or pathway enrichment analysis can be performed on identified key nodes [7].

Graph Neural Networks

In GNNs, during the training process, each model was trained for 200 epochs. The Adam optimizer was used with a learning rate of 0.01 and a weight decay of 5e-4. The purpose of the optimizer is to update the model's parameters to minimize the negative log-likelihood loss. This loss function is commonly used for classification tasks. After training, models were evaluated on a separate test set using metrics like accuracy, precision, recall, and F1-score. Loss curves and confusion matrices were generated for each model, showing the change in loss over training epochs and providing insights into the model's learning performance. Analyzing these metrics, loss curves, and confusion matrices helps researchers understand the performance of graph-based models in classifying data and identifying areas for improvement [8,9].

GAT Architecture

Multi-head attention is a method used to capture various aspects of a graph, with each attention head performing its computation and aggregation. The outputs are concatenated and passed through a linear layer to obtain final node representations. These are then passed through a fully connected output layer and a SoftMax activation function to predict drug-gene association probabilities [8,9].

Graph SAGE architecture

Graph SAGE applies convolutional layers to propagate information between nodes, aggregating information from the node's neighborhood to compute a new representation. The number of convolutional layers and the size of the hidden layers are also important hyperparameters. The aggregated neighborhood vectors are then passed through a fully connected output layer followed by a SoftMax activation function to predict drug-gene association probabilities. Common hyperparameters for training a Graph SAGE model include learning rate, batch size, number of training epochs, dropout, weight decay (L2 regularization), and evaluation metrics like accuracy, precision, recall, and F1-score. These hyperparameters are tuned through a grid or random search to find the optimal combination for the specific task [10,11].

## Results

**Figure 1:**
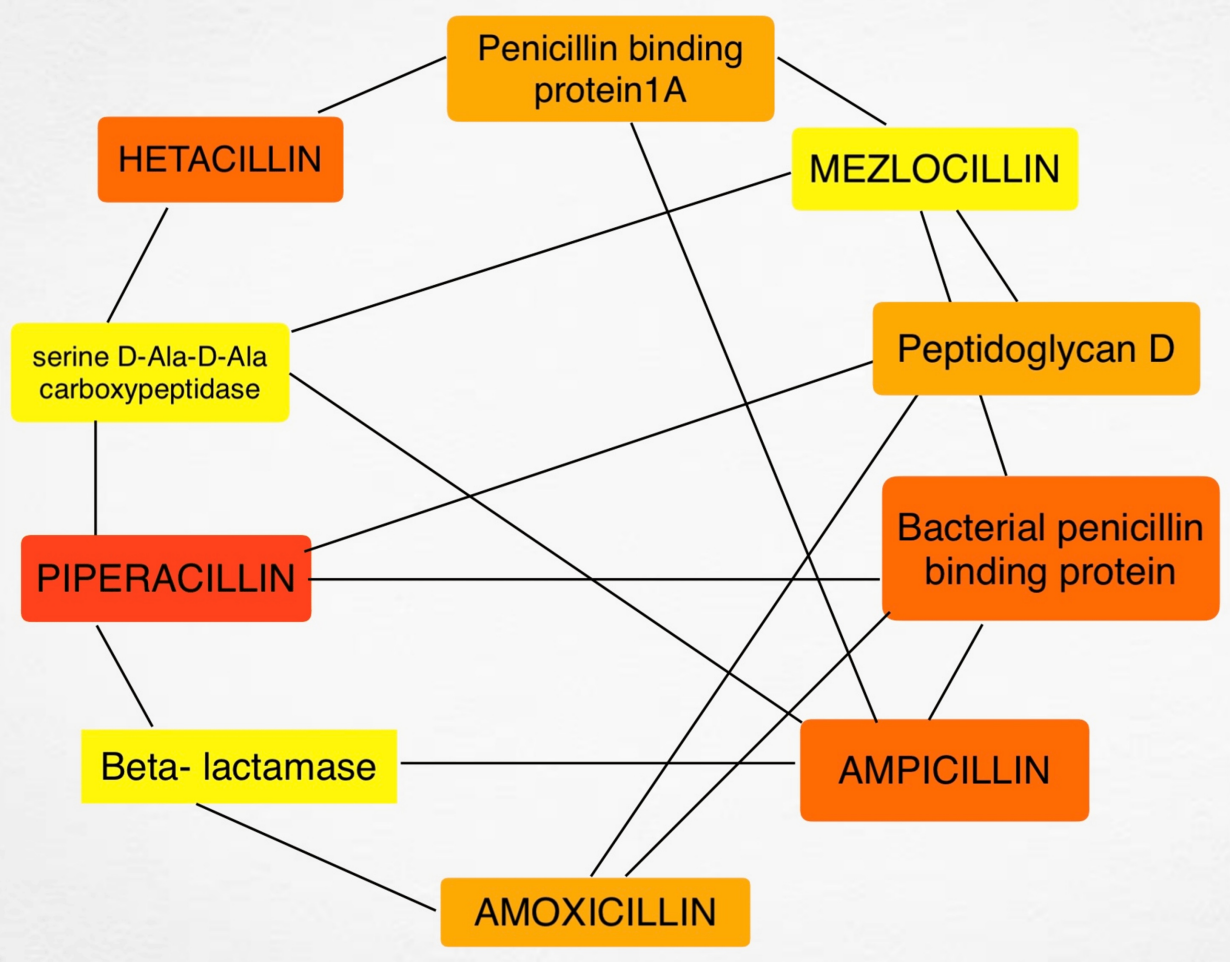
The top hub drug and genes associated with beta-lactamases

The drug-gene association network has 57 nodes, 79 edges, an average number of neighbors, six longest shortest paths, three shortest paths, and 2.730 characteristic path lengths. The clustering coefficient measures the degree to which nodes cluster, while network density represents the proportion of actual connections out of the total possible connections. Network heterogeneity measures the degree to which node degrees are distributed unevenly, while network centralization measures the degree to which the network is centralized around a few highly connected nodes. The network has two separate components, or clusters. These metrics provide insight into the drug-gene association network's structure, organization, and connectivity, helping to understand its properties and potential biological implications as shown in Figure 1.

**Figure 2:**
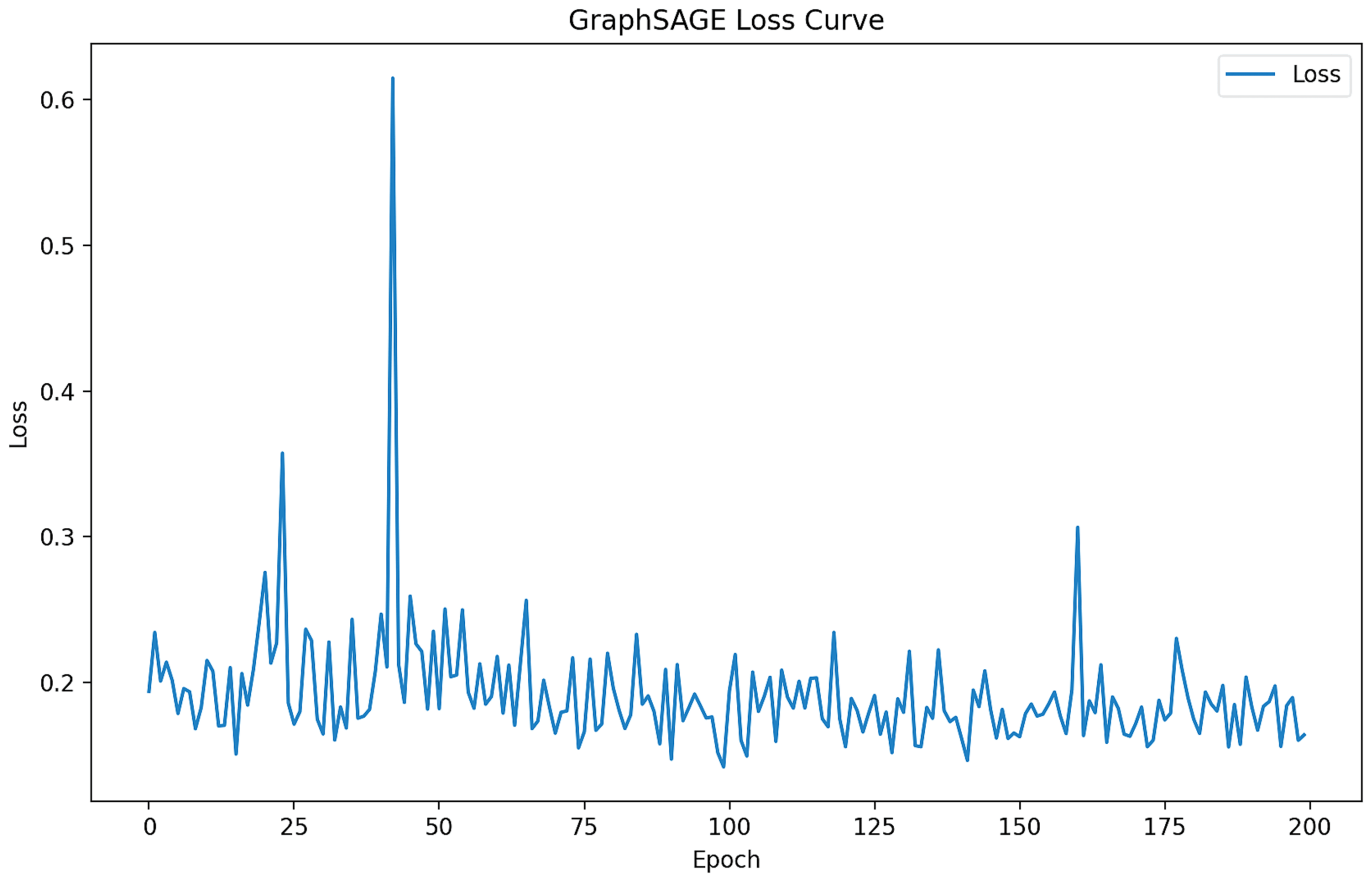
Graph SAGE loss curve SAGE: Sample and Aggregation

**Figure 3:**
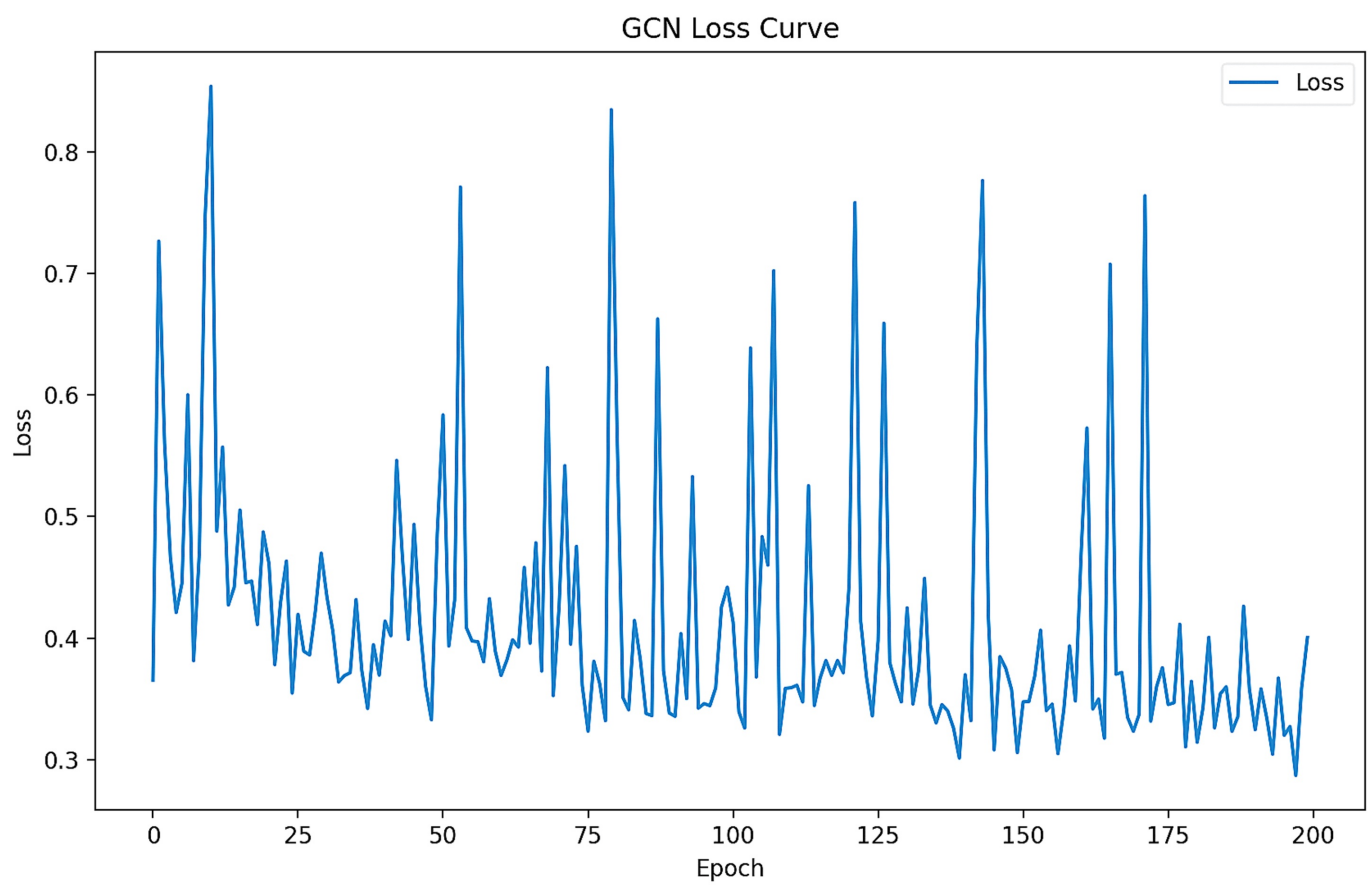
GCN loss curve GCN: Graph Convolutional Network

**Figure 4:**
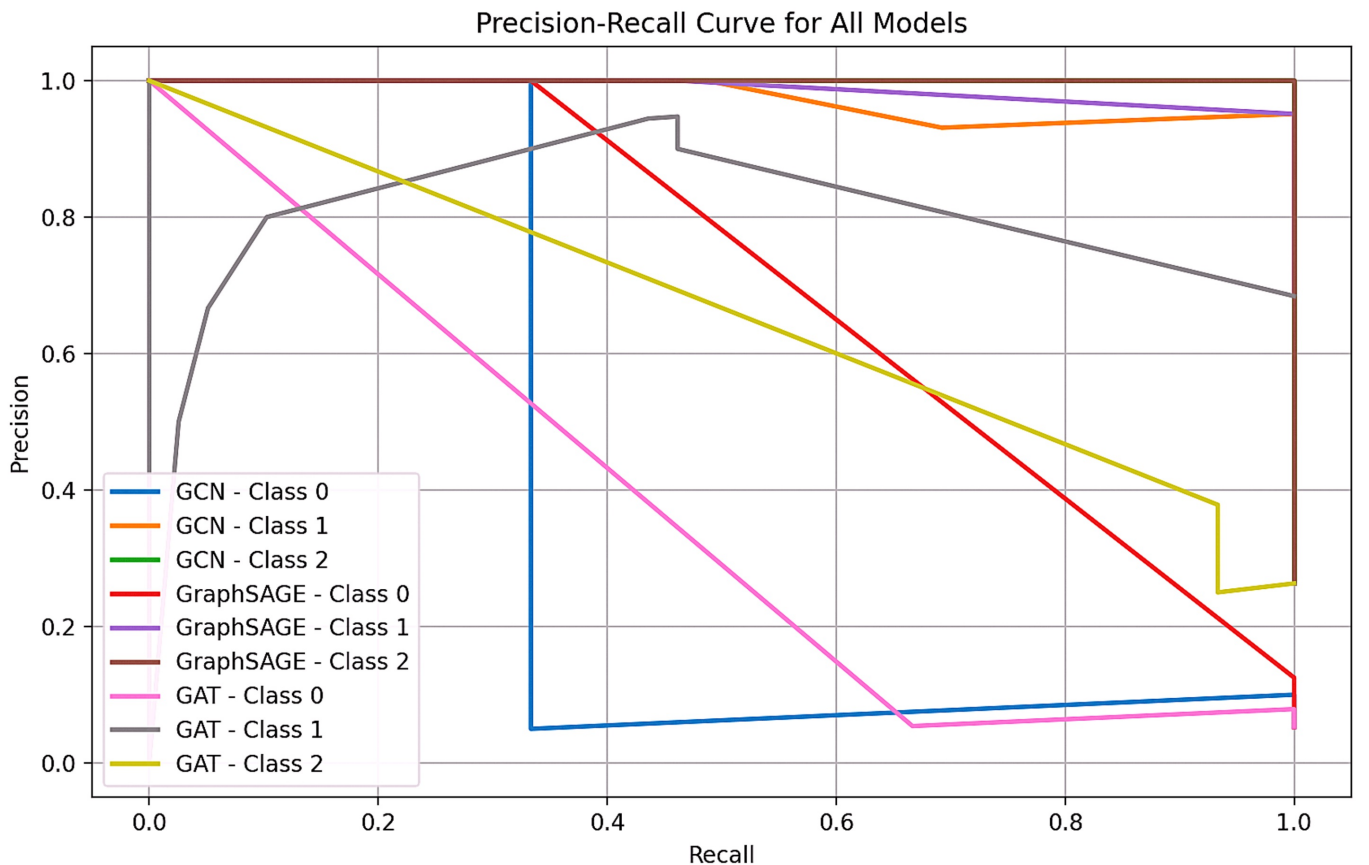
Precision-recall curve for multiple models

GCN and Graph SAGE show high accuracy, precision, recall, and F1-score of 0.94, indicating their effectiveness in predicting drug-gene associations for ESBLs. GAT's performance metrics are lower than GCN and Graph SAGE, with an accuracy of 0.68, precision of 0.47, recall of 0.68, and F1-score of 0.56, suggesting that it may not be as effective in capturing drug-gene relationships as shown in Figures 2-4 and Table 1.

**Table 1:** GCN and Graph SAGE effectively predict drug-gene associations for extended-spectrum beta-lactamases with high accuracy, precision, recall, and F1-score. At the same time, GAT's performance metrics are lower, suggesting it may be less effective GCN: Graph Convolutional Network; SAGE: Sample and Aggregation; GAT: Graph Attention Network

Model	Accuracy	Precision	Recall	F1-score
GCN	0.9473684	0.8984962	0.947368	0.9220273
Graph SAGE	0.9473684	0.8984962	0.947368	0.9220273
GAT	0.6842105	0.468144	0.684211	0.5559211

Figure 2 shows the loss curve for a Graph SAGE model during training, showing fluctuations over epochs, stabilization after initial fluctuations, and final performance hovering around 0.2 by the end of 200 epochs. This visualization is crucial for assessing machine learning model training, helping identify overfitting or underfitting issues, and is essential for assessing the training process of machine learning models.

Figure 3 shows a graph illustrating a GCN loss curve during training. It shows the Y-axis (Loss) and X-axis (Epoch) axes, with the "GCN Loss Curve" labelled for tracking the loss for a GCN. The graph shows fluctuations in loss value over the epochs, with peaks and troughs. The trend suggests a gradual decrease in loss, suggesting the model is learning over time, although some spikes indicate instability.

Figure 4 shows a precision-recall curve for multiple models, comparing their performance across different classes. The graph features an x-axis for recall and a y-axis for precision. The colored lines correspond to different models and class combinations, illustrating the precision and recall trade-offs. The curves indicate the precision and recall of each model, with a curve closer to the top right corner (1,1) indicating better performance. The precision-recall curve is a valuable tool for comparing the effectiveness of machine learning models in classifying instances across different classes. GCN and Graph SAGE perform similarly across all classes, with their lines often overlapping. Both GCN and Graph SAGE show excellent performance for Class 1 and Class 2, reaching close to the top-right corner. The GAT model underperforms compared to GCN and Graph SAGE, especially for Class 2. All models struggle with Class 0, as the curves closer to the bottom-left corner indicate.

## Discussion

Gram-negative anaerobic bacteria are increasingly identified as pathogens and are becoming resistant to β-lactam drugs, posing a challenge for physicians [2]. The β-lactamase enzyme, produced by soil and pathogenic bacteria, interferes with antibiotic activity. The rampant use of antibiotics increases resistance, especially in oral flora, necessitating enzyme testing to prevent prescription failure. A previous study found that 26% of periodontitis patients were ESBL producers, with Bacteroides fragilis and Fusobacterium species being the most common. It underscored the need for antibiotic resistance testing and emergency drug selection [2,3]. A recent study screened supragingival plaque samples from healthy adults with CTX-M-producing E. coli fecal carriage for ESBL-producing bacteria and genes. No ESBL-producing bacteria or genes were found, except for one instance of Rahnella aquatilis carrying the bla RAHN-1/2 gene [4]. This suggests that CTX-M-producing bacteria are uncommon in healthy adults' oral plaque. This suggests that the presence of CTX-M-producing bacteria in oral plaque is rare, but a low prevalence of oral ESBL carriage in healthy adults or specific patient groups cannot be ruled out.

ESBLs are enzymes produced by certain bacteria that confer resistance to various beta-lactam antibiotics, including penicillins. This study identified top hub drug ESBLs that genes like blaCTX-M, blaTEM, and blaSHV can encode. One key resistance mechanism is the production of beta-lactamase enzymes, which cleave the beta-lactam ring of beta-lactam antibiotics, rendering them inactive. This is particularly relevant in bacterial penicillin-binding proteins involved in cell wall synthesis. ESBL-producing bacteria can contribute to developing and progressing infections in periodontal diseases, which involve inflammation and infection of tooth-supporting structures [2]. ESBL-producing bacteria hinder the efficacy of beta-lactam antibiotics, causing persistent infections. Further research is needed to understand ESBL-mediated resistance in periodontal diseases and develop effective treatments.

Graph SAGE architecture performs neighborhood sampling to efficiently process large graphs, with the size of the neighborhood and the number of sampled neighborhoods being important hyperparameters [8]. GAT uses a self-attention mechanism to learn the importance of each node's neighbors in aggregating information [9]. It assigns attention coefficients based on the compatibility of their feature representations. GAT uses a shared attention mechanism to compute attention coefficients, which are then aggregated using the attention coefficients assigned by the attention mechanism [10,11].

GCN and Graph SAGE [12,13] effectively predict drug-gene associations for ESBLs. Still, improvements include incorporating multi-omics data, applying transfer learning techniques, developing interpretable models, and establishing benchmarks. Currently, the data on drug-gene associations is limited, making it difficult to interpret the underlying reasons for their predictions [14]. A previous study introduced DRPreter, a model that predicts anticancer drug response using GNNs. It divides cell-line graphs based on biological pathways and uses a type-aware transformer to identify relationships between drug paths [15].

Limitations and future scope

Future research should focus on increasing the availability of high-quality data and developing standardized datasets for drug-gene association prediction. Overfitting, where a model performs well on training data but fails to generalize to unseen data, should be mitigated through data augmentation, regularization techniques, and model selection based on cross-validation [16]. Ethical considerations are also crucial in applying drug-gene association prediction models, particularly in precision medicine and personalized treatment [17]. Future research should address potential biases and assess the fairness and transparency of these models to ensure their responsible use in periodontal clinical decision-making. Validation and reproducibility of GCN, Graph SAGE, and similar models on independent datasets are crucial for assessing their generalizability and reproducibility, while open-source implementations and public data accessibility enhance transparency.

## Conclusions

GCN and Graph SAGE have superior performance in predicting drug-gene associations for ESBLs, outperforming GAT in accuracy, precision, recall, and F1-score. Compared to ESBLs, both GCN and Graph SAGE demonstrate excellent performance with accuracy, precision, recall, and an F1-score of 0.94. These results indicate that GCN and Graph SAGE are highly effective in predicting drug-gene associations related to ESBLs. GAT's performance metrics are lower, with lower accuracy, precision, recall, and F1-score, suggesting that it may not be as effective in capturing drug-gene relationships for ESBLs. At the same time, GCN and Graph SAGE show superior performance. However, improvements include incorporating more data sources, applying transfer learning techniques, and developing interpretable models. Limited high-quality data availability also poses challenges. To improve the reliability and generalizability of drug-gene association prediction models, strategies like data augmentation, regularization, and cross-validation are needed. Ethical considerations are crucial in precision medicine and personalized treatment. Future research should address biases and ensure fairness for responsible decision-making in periodontal clinical practice. Validating and reproducing models on independent datasets is essential, and open-source implementations and public data accessibility enhance transparency.
